# Evolutionary Conservation of Divergent Pro-Inflammatory and Homeostatic Responses in Lamprey Phagocytes

**DOI:** 10.1371/journal.pone.0086255

**Published:** 2014-01-20

**Authors:** Jeffrey J. Havixbeck, Aja M. Rieger, Michael E. Wong, Michael P. Wilkie, Daniel R. Barreda

**Affiliations:** 1 Department of Biological Sciences, University of Alberta, Edmonton, Alberta, Canada; 2 Department of Agricultural, Food and Nutritional Science, University of Alberta, Edmonton, Alberta, Canada; 3 Department of Biology, Wilfrid Laurier University, Waterloo, Ontario, Canada; Chang Gung University, Taiwan

## Abstract

In higher vertebrates, phagocytosis plays a critical role in development and immunity, based on the internalization and removal of apoptotic cells and invading pathogens, respectively. Previous studies describe the effective uptake of these particles by lower vertebrate and invertebrate phagocytes, and identify important molecular players that contribute to this internalization. However, it remains unclear if individual phagocytes mediate internalization processes in these ancient organisms, and how this impacts the balance of pro-inflammatory and homeostatic events within their infection sites. Herein we show that individual phagocytes of the jawless vertebrate *Petromyzon marinus* (sea lamprey), like those of teleost fish and mice, display the capacity for divergent pro-inflammatory and homeostatic responses following internalization of zymosan and apoptotic cells, respectively. Professional phagocytes (macrophages, monocytes, neutrophils) were the primary contributors to the internalization of pro-inflammatory particles among goldfish (*C. auratus*) and lamprey (*P. marinus*) hematopoietic leukocytes. However, goldfish showed a greater ability for zymosan phagocytosis when compared to their jawless counterparts. Coupled to this increase was a significantly lower sensitivity of goldfish phagocytes to homeostatic signals derived from apoptotic cell internalization. Together, this translated into a significantly greater capacity for induction of antimicrobial respiratory burst responses compared to lamprey phagocytes, but also a decreased efficacy in apoptotic cell-driven leukocyte homeostatic mechanisms that attenuate this pro-inflammatory process. Overall, our results show the long-standing evolutionary contribution of intrinsic phagocyte mechanisms for the control of inflammation, and illustrate one effective evolutionary strategy for increased responsiveness against invading pathogens. In addition, they highlight the need for development of complementary regulatory mechanisms of inflammation to ensure continued maintenance of host integrity amidst increasing challenges from invading pathogens.

## Introduction

The immune system has evolved to confer effective protection against infection, driven by continuous interactions between hosts and microbes. The resulting multi-layered system increasingly requires complex cross-regulatory systems [1], [2]. At the core of these responses, phagocytosis continues to fill increasing roles as an inducer and regulator of host immunity. Internalization of pathogens by phagocytes leads to stimulation of potent killing mechanisms such as the production of reactive oxygen species (ROS) that have evolved to degrade and kill foreign invaders and contribute to downstream adaptive mechanisms [3]. Importantly, the capacity for effective internalization and clearance of apoptotic cells is already well established in developmental pathways of early multi-cellular organisms [4]–[6]. Phagocytic receptors in *Caenorhabditis elegans* (CED-1) and *Drosophila melanogaster* (Draper), for example, drive recognition and internalization of apoptotic corpses and activate downstream processing pathways that are central to morphogenesis and the maintenance of tissue integrity and function [7], [8].

At the site of infection, mammalian phagocytes effectively shape the environmental milieu for destruction of invading pathogens or resolution of tissue inflammation. Pathogen engagement leads to rapid production of pro-inflammatory mediators including the production of reactive oxygen and nitrogen species [9], release of antimicrobial peptides [10], and the secretion of tumor necrosis factor alpha (TNF-α), interferon gamma (IFN-γ) and IL-1 beta (IL-1β) [11]. In contrast, internalization of apoptotic cells initiates the shift towards resolution mechanisms that promote tissue repair and a return to homeostasis once the pathogen has been effectively cleared. This is marked by increases in interleukin 10 (IL-10), transforming growth factor beta 1 (TGF-β1), prostaglandin E2 and platelet activating factor [12]–[14], combined with decreases in pro-inflammatory mediators, including tumor necrosis factor alpha (TNF-α), IL-6, IL-8, IL-12, IL-17, IL-23, leukotriene C4 and thromboxane B2 [13], [15], [16].

We previously showed that phagocytes of teleost fish contributed to both pro-inflammatory and anti-inflammatory (resolution) responses at infectious foci [17]. Like murine phagocytes, they possessed the capacity to balance between these two seemingly contradictory processes. However, teleost phagocytes displayed significant differences *in vivo* with regards to the level of responsiveness to zymosan and apoptotic bodies, the identity of leukocytes infiltrating the infectious site, their rate of infiltration, and the kinetics and strength of resulting antimicrobial responses [17]. The striking evolutionary differences observed in inflammatory control between mice and goldfish provided a platform to investigate these differences down the evolutionary scale in a primordial vertebrate.

In this study, we compared the effects of homeostatic phagocytosis on the regulation of pro-inflammatory responses in goldfish (*Carassius auratus*) and sea lamprey (*Petromyzon marinus*). As one of the earliest vertebrates, along with hagfish (Myxinidae), sea lampreys were an appropriate model to investigate the conservation of this dichotomy. Through the use of zymosan, a pro-inflammatory stimulus known to induce production of reactive intermediates [18]–[24], the impact of apoptotic cells on the production of reactive oxygen species (ROS) was examined. Interestingly, we found notable differences between goldfish and lamprey ROS responses in phagocytes that had internalized both zymosan and apoptotic bodies, though responses in cells that had internalized only zymosan or only apoptotic bodies were largely conserved. This work underscores the importance of phagocytosis as a phylogenetically ancient process essential to the innate immune response.

## Materials and Methods

### Ethics Statement

All animals were maintained according to the guidelines of the Canadian Council on Animal Care, and protocols were approved by the University of Alberta Animal Care and Use Committee (ACUC-Biosciences; protocol numbers 595807 and 706). Both goldfish and lampreys were terminated via cervical dislocations using approved procedures following anaesthetization with tricaine methanesulfonate (TMS-222). All efforts were made to minimize animal stress and to ensure that termination procedures were performed efficiently.

### Animals

Goldfish (*Carassius auratus* L.) 10–15 cm in length were purchased from Mount Parnell (Mercersburg, PA). The fish were held at 18°C in a flow-through water system with constant aeration. Ammocoete larvae (8–11 cm in length) of the sea lamprey (*Petromyzon marinus*) were received from Wilfred Laurier University, previously captured by electrofishing from freshwater streams in New Brunswick, Canada. Lamprey were maintained in sand-lined aquaria with constant aeration at 18°C and fed brewer’s yeast. All animals were housed in the Aquatic Facility of the Department of Biological Sciences, University of Alberta, on a simulated natural photosystem.

### Isolation of Hematopoietic Leukocytes

Goldfish primary kidney leukocytes (PKL) were isolated by maceration of goldfish kidney tissue between two glass slides suspended in incomplete MGFL-15 medium. Lamprey primary typhlosole leukocytes were isolated as previously described [25]. Briefly, larval lampreys were dissected along the ventral side to extract the intestine and the associated typhlosole. Cells were harvested by maceration between two glass slides suspended in one part water and two parts incomplete MGFL-15 medium.

### Light Microscopy Phagocytosis Assay

Latex beads (3 µm; Polysciences) were added at ratios of 1∶1, 5∶1 and 10∶1 (bead: cell) to 1×10^5^ cells in 24 well plates and incubated at 18°C for the indicated times. Cells were stained by Hema3 stain set (Fisher Scientific) and counted by light microscopy. Phagocytic index was calculated based on the number of beads internalized per phagocyte. A minimum of 200 cells were evaluated per sample. As per previous reports, internalization of particles larger than 0.5 µm leads to activation of downstream phagocytic events [26].

### Preparation of Zymosan and Apoptotic Cells

Unlabeled zymosan particles (Molecular Probes) were labeled overnight with 250 ng/mL FITC (Sigma) with continuous shaking at 4°C in carbonate buffer (0.1 M sodium carbonate, 0.1 M sodium bicarbonate; pH 9.6). After staining, zymosan-FITC was washed twice with 1×PBS^−/−^. Unlabeled zymosan particles (Molecular Probes) were labeled overnight with 75 µg/mL allophycocyanin (APC; Sigma) with continuous shaking at 4°C in 1× PBS^−/−^. Apoptotic cells were generated by incubating cells for 24 h in the presence of 10 µg/mL cycloheximide. Treated cells were harvested, washed twice in 1× PBS^−/−^ and stained overnight with 1.5 µg/mL wheat germ agglutinin Alexa Fluor 555 (Molecular Probes). Apoptotic cells were then washed twice in 1× PBS^−/−^. For goldfish experiments, apoptotic cells were generated from catfish 3B11 B cells. For lamprey, apoptotic cells were generated from primary typhlosole leukocytes. We have previously shown that apoptotic cells derived from primary or cell line leukocytes induce equivalent phagocyte responses [17].

### ImageStream Phagocytosis Assay

Latex beads (3 µm; Polysciences), *E. coli* DH5α-GFP or zymosan-FITC were added to 1×10^5^ cells at ratios of 5∶1 and 10∶1 (particles: cells) and incubated for the indicated times. Following incubation the cells were washed twice in 1× PBS^−/−^ and fixed in 1% formaldehyde at 4°C overnight. Phagocytic index was calculated based on the number of particles internalized per phagocyte. Data was acquired on an ImageStream multi-spectral imaging flow cytometer (Amnis) and analyzed using INSPIRE software. At least 10000 cells were acquired. Preliminary experiments indicated that cells remained within their corresponding leukocyte gate following internalization of *E. coli* or zymosan, but not following internalization of 3 µm beads, where internal complexity increased significantly. Development of specific markers for each of these leukocyte subsets should further enhance the characterization of phagocytic events within each of these phagocyte subsets.

### Respiratory Burst Assay

This assay was performed as previously described with minor modifications [27], [28]. Cells were harvested and collected into 5 mL polystyrene round bottom tubes (BD Falcon). Dihydrorhodamine (DHR, Molecular Probes) was added to the cells at a final concentration of 1 µM and incubated for 5 min to allow the cells to take up the DHR. Phorbol 12-myristate 13-acetate (PMA; Sigma) was then added at a final concentration of 10 ng/mL. Cells were further incubated for 30 minutes to allow oxidation of the DHR. All samples were appropriately staggered with respect to timing to accommodate for the transient state of oxidized DHR fluorescence. DHR fluorescence was not quenched by the presence of other fluorochromes, including the wheat germ agglutinin or APC labels on phagocytosed particles (data not shown).

### Analysis

ImageStream data was analyzed using IDEAS software (Amnis), as previously described [27]. Statistics were performed by Students’ T-test using Prism 6 software (GraphPad Prism).

## Results

### Goldfish Phagocytes Display a Greater Capacity for the Internalization of Pro-inflammatory Particles than Lamprey Phagocytes

**Figure 1:**
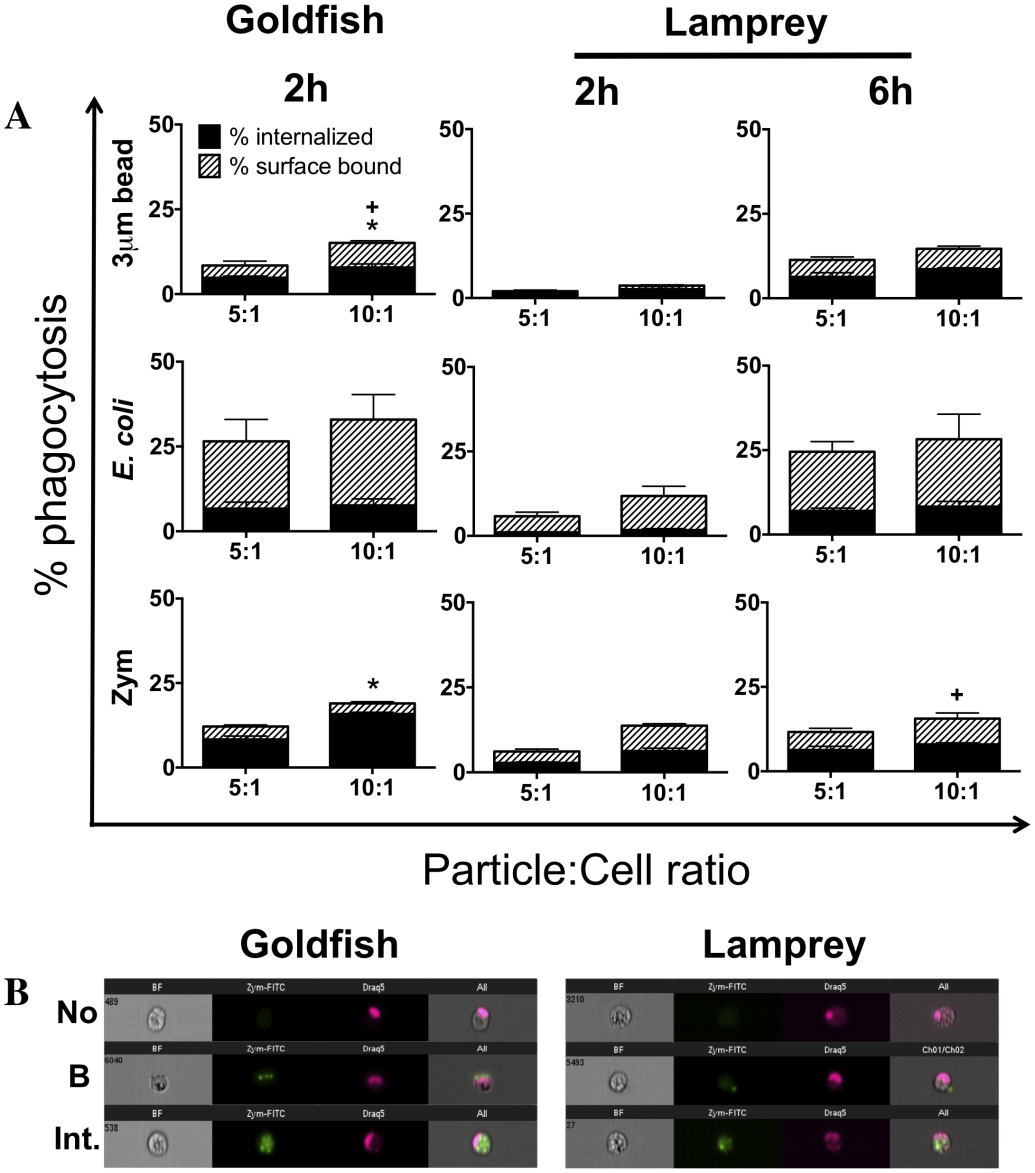
Phagocytosis of different target particles by goldfish and lamprey primary leukocytes. (A) Goldfish primary kidney leukocytes (PKL) or lamprey primary typhlosole leukocytes (PTL) were incubated with 3 µm YG latex beads, *E. coli* DH5α-GFP, or zymosan-FITC at the indicated concentrations for the specified times. Cells were then fixed and phagocytosis was quantified by flow cytometry. Grey bars represent percent internalized. Hatched white bars represent percent surface bound. For all n = 4, over 2 examined over a minimum of two independent experiments. * p<0.05 for % internalized,+p<0.05 for % surface bound- between 10∶1 and 5∶1 particle to cell ratios in each graph. (B) Representative images of no internalization (No), surface bound (B), and internalized beads (Int.) from ImageStream MkII flow cytometer (Amnis).

As a first step in the characterization of differences between the contributions of agnathan (jawless fish) and teleost (bony fish) phagocytes to the control of inflammation, we compared the phagocytic capacity of primary leukocytes from sea lamprey typhlosole and goldfish kidney. These corresponded to the primary hematopoietic tissues of the animals examined and provided sufficient numbers of their primary phagocyte populations for *ex vivo* examination [29]–[32]. Phagocytosis of three commonly used model particles was examined: i) 3 µm latex beads, ii) *E. coli,* and iii) zymosan (Figure 1A). Phagocytosis was assessed by imaging flow cytometry, which allowed discrimination of bound and internalized particles (Figure 1B; [27]). From the outset, our experiments indicated differences in the efficiency of phagocytosis for goldfish and lamprey leukocytes *ex vivo*. Two hours was sufficient to examine basal levels of phagocytosis in goldfish (Figure 1A). In contrast, six hours were required to achieve equivalent levels of phagocytosis among sea lamprey leukocytes. This was further corroborated by a conventional light microscopy phagocytosis assay, and determination of optimal respiratory burst levels (Figure S1 and S2, respectively). Results were not consistent across the three pro-inflammatory particles examined. Lamprey leukocytes continued to display lower levels of zymosan phagocytosis that goldfish even after six hours of incubation (Figure 1A, bottom row).

**Figure 2:**
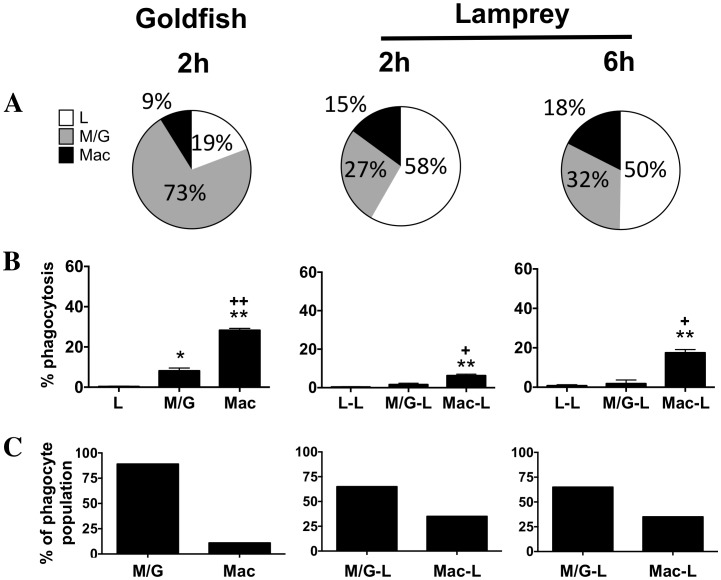
Macrophages are the dominant phagocyte of goldfish and lamprey primary hematopoietic tissues. (A) Goldfish PKL and lamprey PTL were incubated with zymosan (5∶1 ratio) for the specified times. The total leukocyte pool was broken down into cellular subpopulations based on morphology and flow cytometry forward and side scatter parameters. (B) The percent of phagocytic cells from each subpopulation previously determined in (A). * p<0.05 and ** p<0.01 compared to lymphocytes;+p<0.05 and++p<0.01 compared to monocytes/granulocytes. (C) The percent of monocytes/granulocytes and macrophages that make up the professional population of phagocytes. For all n = 4, examined over a minimum of two independent experiments.

A greater capacity for phagocytosis of zymosan suggested an increased ability of goldfish phagocytes to effectively mount antimicrobial defenses against fungal pathogens. We wondered whether this difference was associated with differences in the relative abundance of distinct phagocyte groups within the hematopoietic leukocyte pool and/or differences in their phagocytic capacity compared to those in lamprey (Figure S3). We focused on the granulocyte, monocyte, and macrophage populations as the classical professional phagocytes of fish, and the lymphocyte population as the newest members of the phagocyte group [33]–[35]. Although leukocyte populations in lamprey displayed similar cellular characteristics to those of goldfish based on size, morphology and internal complexity the range of reagents available to help define various subsets is still limited. As such, they remain as lymphocyte-like, granulocyte-like, monocyte-like and macrophage-like cells. For goldfish, granulocytes/monocytes represented the majority of the leukocytes isolated from kidney hematopoietic tissues (73%), with smaller contributions from macrophage and lymphocyte populations (19% and 9% respectively; Figure 2A). For the lamprey, lymphocyte-like cells comprised the majority of the total typhlosole leukocyte pool (50%; Figure 2A, 6 h). Granulocyte/monocyte and macrophage-like cells followed and contributed approximately 32% and 18%, respectively. Evaluation of phagocytosis indicated that macrophage and macrophage-like cells were the primary mediators of zymosan internalization in goldfish and lamprey, respectively (Figure 2B). However, these cells represented different proportions of the hematopoietic phagocyte pool that internalized zymosan in goldfish and lamprey (11% and 35%, respectively; Figure 2C). Further, they also displayed a differential ability to internalize zymosan (28% and 17% phagocytosis, respectively; Figure 2B). Despite their lower abundance within the hematopoietic tissues examined, goldfish macrophages showed a marked greater capacity to internalize zymosan, which contributed to overall greater levels of zymosan phagocytosis for goldfish kidney leukocytes (16% in goldfish versus 6% in lampreys; Figure 1A). The increased relative efficacy for zymosan internalization in goldfish was not limited to the macrophage population. Despite their lower contribution to the internalization of zymosan, monocytes/granulocytes also displayed a four-fold greater capacity for zymosan phagocytosis in goldfish compared to the monocyte/granulocyte-like pool in lamprey (8% versus 2%, respectively; Figure 2B). Thus, professional phagocytes (macrophages, monocytes, neutrophils) are the primary contributors to the internalization of zymosan in both goldfish and lamprey hematopoietic leukocytes; however, our teleost phagocytes displayed a significantly greater ability for zymosan phagocytosis when compared to their agnathan counterparts. Notably, the low levels of phagocytosis observed among goldfish lymphocytes and lamprey lymphocyte-like cells in these experiments (0.27% and 0.8%, respectively) may stem from their limitation for internalization of larger particles (2.5–3 µm for zymosan) and not an overall inability for phagocytosis. Alternatively, this may be associated with a reduced capacity to interact with zymosan. Indeed, evaluation of the capacity for *E. coli* internalization among the hematopoietic leukocyte subsets examined showed increased levels of phagocytosis among lamprey lymphocyte-like cells when compared to zymosan. Whereas 0.8% of lamprey lymphocyte-like cells showed zymosan internalization under the experimental conditions tested, 2.3% showed *E. coli* uptake. Given the levels of bacterial uptake observed, this attributed 13% of the *E. coli* phagocytosis to the lymphocyte-like population. As lymphocyte-like cells corresponded to approximately 50% of the leukocyte population examined in lamprey (Figure 2A), this points to a potentially significant contribution by these cells to bacterial phagocytosis.

### Goldfish Phagocytes Display Prominent Respiratory Burst Responses, even when Apoptotic Cells are Internalized

**Figure 3:**
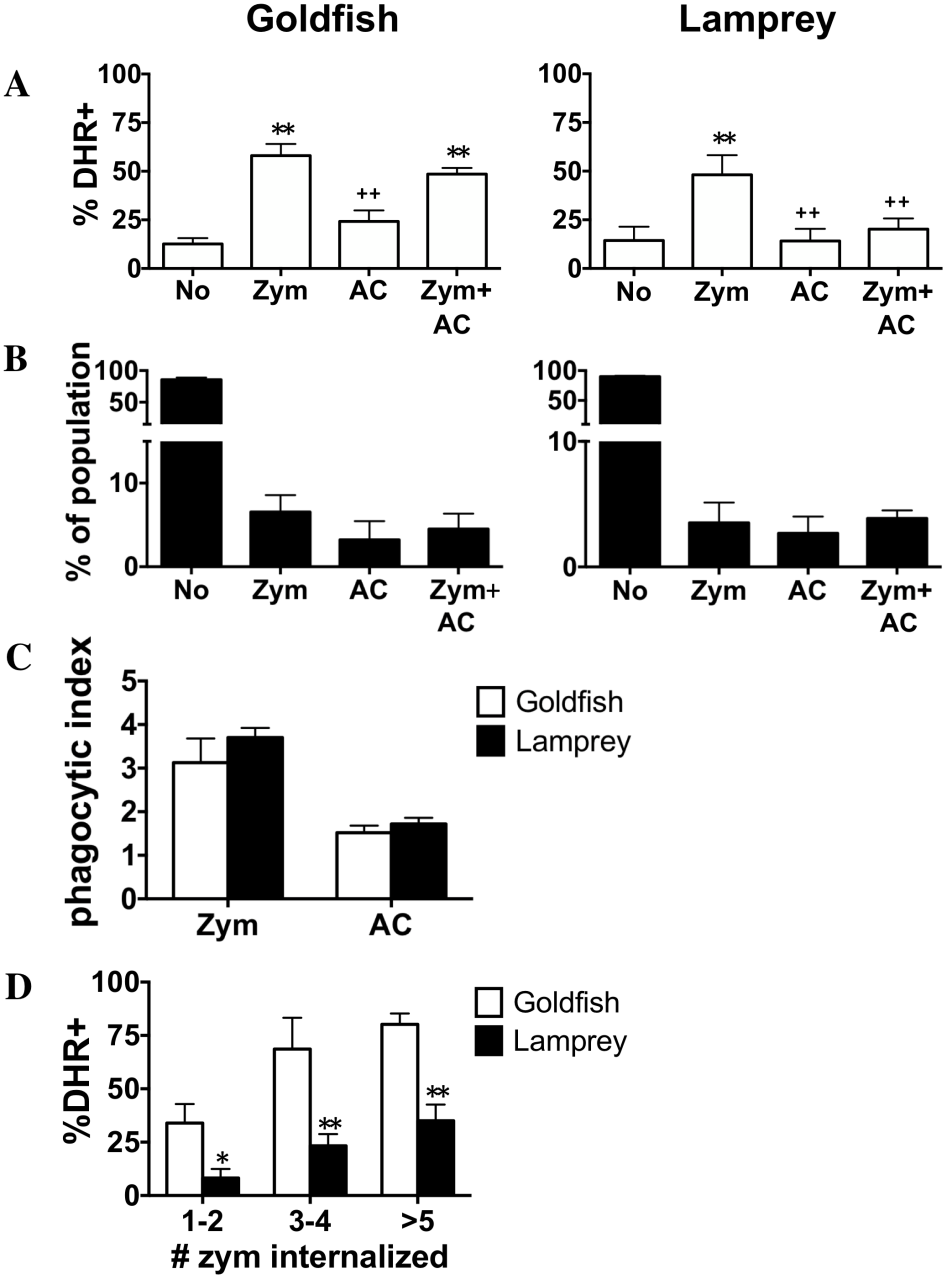
Divergent pro-inflammatory and homeostatic responses of lamprey and goldfish phagocytes. Goldfish PKL and lamprey PTL were incubated with both zymosan and apoptotic cells (5∶1 ratio for each) for 2 h and 6 h, respectively. (A) Respiratory burst (measured as % DHR positive) was then analyzed based on phagocytic capacity across the four resulting sub-populations: non-phagocytic cells, phagocytes containing only zymosan, phagocytes containing only apoptotic cells, and phagocytes that contain both. (B) The percent of total population found in each of the four sub-populations of (A); no internalization, zymosan only, apoptotic cells only, zymosan and apoptotic cells. * p<0.05 and ** p<0.01 compared to No;+p<0.05 and++p<0.01 compared to Zym. (C) The phagocytic index of the Zym+AC group in (A). (D) Respiratory burst analyzed according to the number of zymosan particles internalized in the Zym+AC group. * p<0.05 and ** p<0.01 compared to goldfish. No- no internalized particle; AC- apoptotic cells; Zym- zymosan. For all n = 4, examined over a minimum of two independent experiments.

We previously determined that teleost phagocytes, like those of mice, displayed the capacity for divergent pro-inflammatory and homeostatic responses, following internalization of zymosan and apoptotic cells, respectively [17]. Further, internalization of zymosan, apoptotic cells, or both by individual phagocytes contributed differentially to the modulation of antimicrobial inflammatory responses [17]. Accordingly, we investigated whether or not a similar mechanism of inflammatory control at the level of the individual phagocyte was already displayed in the lamprey. As expected, zymosan internalization resulted in an increase in the level of the respiratory burst response in both goldfish and lamprey (Figure 3A). Conversely, internalization of apoptotic cells led to a significant reduction in ROS production in both species. Thus, our results indicated that sea lamprey phagocytes were already capable of mediating divergent pro- and anti-inflammatory responses. Interestingly, when we analyzed the level of ROS production in cells that had internalized both zymosan and apoptotic cells, we saw a striking difference between goldfish and lamprey phagocytes. Goldfish phagocytes that had internalized both zymosan and apoptotic cells showed equivalent levels of ROS production compared to those that internalized zymosan alone (p = 0.88; Figure 3A). In sharp contrast, lamprey phagocytes that internalized apoptotic cells displayed low levels of ROS production even when zymosan was co-internalized (Figure 3A). Given that the proportion of phagocytes internalizing zymosan, apoptotic cells, or both were consistent between goldfish and lampreys (Figure 3B), this may reflect a greater drive for induction of inflammatory antimicrobial responses in goldfish in response to zymosan.

Examination of the Zym+AC group presented an opportunity to examine the mechanism(s) by which individual phagocytes regulate inflammatory processes following internalization of pro-inflammatory or homeostatic particles. One possibility for the greater capacity of goldfish phagocytes to display robust respiratory burst responses was that individual goldfish phagocytes internalized a greater number of zymosan particles. Alternatively, they may have internalized fewer apoptotic cells. However, our results showed that goldfish and lamprey primary hematopoietic tissue phagocytes did not display a differential capacity to internalize zymosan or apoptotic cells (Figure 3C). Instead, subsequent analysis revealed that goldfish phagocytes were more responsive to zymosan internalization than those in lamprey, as evidenced by the relative strength of respiratory burst responses (Figure 3D). When the number of internalized apoptotic cells was kept constant, relative increases in the internalization of zymosan (1 apoptotic cell with 1–2, 3–4, or >5 zymosan particles) translated to a dramatic increase in ROS production in goldfish phagocytes (Figure 3D). In contrast, lamprey phagocytes displayed much less prominent respiratory burst responses in all groups examined when compared to that of zymosan alone. Thus, internalization of even a single apoptotic cell impaired the ability of lamprey, but not goldfish phagocytes, to mount robust pro-inflammatory respiratory burst antimicrobial responses when a phagocyte encountered both pro-inflammatory and homeostatic particles, as would commonly occur within an infection site.

**Figure 4:**
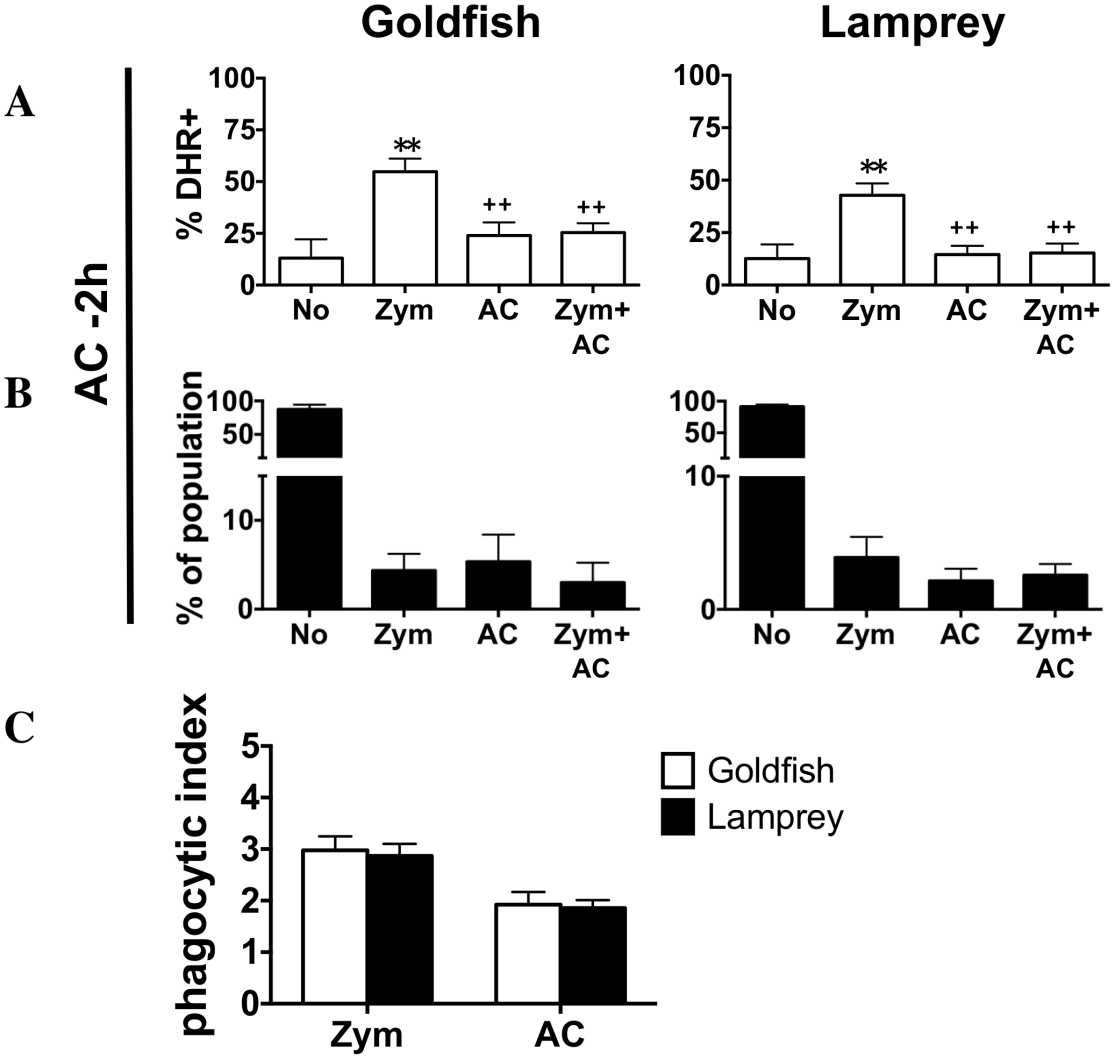
Effect of pre-incubation with zymosan and apoptotic cells on respiratory burst responses of individual phagocytes. Goldfish PKL and lamprey PTL were incubated with both zymosan and apoptotic cells (5∶1 ratio for each) for 2 h and 6 h, respectively. (A) To investigate the effects of pre-incubation with apoptotic cells, apoptotic cells were added 2 h prior to zymosan. Respiratory burst (measured as % DHR positive) was then analyzed based on phagocytic capacity across the four resulting sub-populations: non-phagocytic cells, phagocytes containing only zymosan, phagocytes containing only apoptotic cells, and phagocytes that contain both. (B) The percent of total population found in each of the four sub-populations of (A); no internalization, zymosan only, apoptotic cells only, zymosan and apoptotic cells. (C) The phagocytic index of the Zym+AC group in (A). For all n = 4, examined over a minimum of two independent experiments. * p<0.05 and ** p<0.01 compared to No;+p<0.05 and++p<0.01 compared to Zym.

Importantly, the increased strength in goldfish phagocyte respiratory burst responses described above did not preclude their effective inhibition by homeostatic stimuli. Pre-incubation of primary leukocytes with apoptotic cells (−2 h) was sufficient to impair the respiratory burst response of goldfish leukocytes (Zym+AC group; Figure 4A). The relative proportion of phagocytes internalizing zymosan, apoptotic cells, or both remained consistent between goldfish and lamprey (Figure 4B), as was observed when both particles were added at the same time (Figure 3B). Further, the phagocytic index of the Zym+AC population remained constant in both organisms even when apoptotic cells were added two hours prior to zymosan (Figure 4C). Thus, despite the greater ability of goldfish phagocytes to mount robust respiratory burst responses to zymosan, a model develops whereby goldfish phagocytes can remain as important contributors to the resolution phase of inflammation following early induction of potent antimicrobial pro-inflammatory mechanisms.

The experiments presented also showcase the responses of individual phagocytes amidst a microenvironment that contains mixed populations of phagocytes that have internalized zymosan, apoptotic cells, both particle types or none. Both goldfish and lamprey displayed marked compartmentalization in the activation of respiratory burst responses among phagocyte subgroups following the internalization of pro-inflammatory and/or homeostatic particles. As such, these results highlight the importance of intrinsic mechanisms of inflammation control at the level of the individual phagocyte in both of these animal groups.

### Decreased Goldfish Phagocyte Sensitivity to Apoptotic Cells Contributes to Pronounced Antimicrobial ROS Production but Decreased Efficacy in Leukocyte Homeostatic Responses

**Figure 5:**
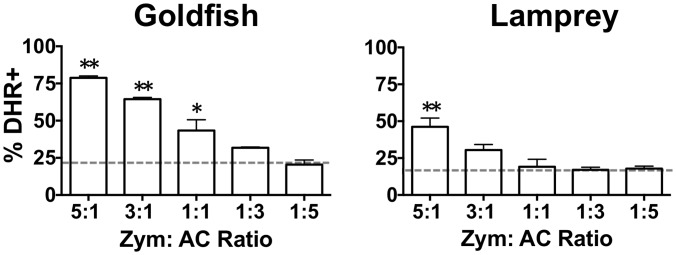
Respiratory burst responses of goldfish and lamprey hematopoietic leukocytes following zymosan and apoptotic cell stimulation. Goldfish PKL and lamprey PTL were incubated with both zymosan and apoptotic cells (5∶1 ratio for each) for 2 h and 6 h, respectively. To examine the effects of a dose response, zymosan and apoptotic cells were added at varying concentrations. Respiratory burst (measured as % DHR positive) was then analyzed for the entire leukocyte population. For all n = 6, examined over a minimum of two independent experiments. Grey dashed line represents the respiratory burst of phagocytes internalizing only AC cells. * p<0.05 and ** p<0.01 compared to grey dashed line (% DHR of phagocytes internalizing only apoptotic cells).

Examination of ROS production amidst mixed cellular populations allowed us to assess the broader impact of phagocyte responses on the total leukocyte pool. Specifically, we sought to determine how the differential sensitivity to pro-inflammatory and homeostatic particles for goldfish and lamprey phagocytes identified above contributed to the control of antimicrobial respiratory burst responses. Varying ratios of zymosan to apoptotic cells (pro-inflammatory and homeostatic particles, respectively) were co-incubated with goldfish and lamprey total hematopoietic leukocyte isolates prior to evaluation of respiratory burst responses (Figure 5). The goal was to mimic the natural shift that occurs at an infection site, where phagocytes initially encounter greater proportions of pro-inflammatory particles (pathogens) followed by increasing proportions of homeostatic particles (apoptotic cells), which ultimately contribute to the activation of tissue repair mechanisms and a return to homeostasis [13], [36], [37]. Consistent with a higher capacity for pro-inflammatory ROS production, goldfish leukocytes displayed greater efficacy in the induction of respiratory burst responses than those of lamprey. However, examination of decreasing Zym to AC ratios showed that this further translated into a lower sensitivity to apoptotic cell homeostatic signals in goldfish compared to lamprey. Goldfish leukocytes required a three-fold greater amount of apoptotic cells than zymosan (1∶3 Zym to AC group, Figure 5) to reach basal levels of ROS production. In contrast, lamprey leukocytes reached these levels even when zymosan out-numbered apoptotic cells three to one (i.e. not statistically significant to basal levels of ROS production). For our experiments, basal levels of ROS production (grey dashed line, Figure 5) were derived from those phagocytes that exclusively internalized apoptotic cells (Figure 3A). As such, our results suggest that the reduced sensitivity of goldfish phagocytes to apoptotic cells translates to overall greater capacity for induction of antimicrobial respiratory burst responses but also a decreased efficacy in leukocyte homeostatic mechanisms that attenuate this pro-inflammatory process.

## Discussion

Phagocytosis is a phylogenetically ancient innate defense strategy that has served as an important platform for the evolution of mechanisms of inflammation control [13], [17], [37]–[39]. Previous studies from our lab focused on the divergent responses of teleost and murine phagocytes following internalization of pathogen-derived and homeostatic particles [17]. In the present study, we show that individual phagocytes of the jawless vertebrate *Petromyzon marinus* (sea lamprey), like those of teleost fish and mice, display the capacity for divergent pro-inflammatory and homeostatic responses. Phagocytes isolated from sea lamprey typhlosole and goldfish kidney hematopoietic tissues were able to internalize a range of particles including latex beads, *E. coli*, and zymosan. However, goldfish leukocytes displayed greater efficiency in the internalization of these pro-inflammatory particles. Examination of phagocytic subsets at the single cell level indicated that, for zymosan, macrophages displayed the greatest capacity of internalization despite representing a significantly lower proportion of the phagocytes within the hematopoietic leukocyte pool. Thus, although professional phagocytes (macrophages, monocytes, neutrophils) were important contributors to the internalization of zymosan in both goldfish and lamprey hematopoietic leukocytes, teleost phagocytes displayed a significantly greater ability for zymosan phagocytosis when compared to their agnathan counterparts.

Although we found some conservation of phagocyte functional responses between lamprey and goldfish, we also saw significant differences in the level and control of these responses to pro-inflammatory and homeostatic stimuli. Goldfish phagocytes that had internalized both zymosan and apoptotic cells showed ROS levels similar to those induced in cells that internalized only zymosan. In sharp contrast, lamprey phagocytes that internalized both stimuli displayed basal levels of ROS production. Goldfish and lamprey primary hematopoietic tissue phagocytes did not display a differential capacity to internalize zymosan or apoptotic cells - the phagocytic index of each particle was similar in phagocytes derived from each animal. Instead, our results suggest that goldfish phagocytes are more responsive to internalized zymosan than those in lamprey based on the relative strength of respiratory burst responses observed. Internalization of even a single apoptotic cell impaired the ability of lamprey, but not goldfish phagocytes, to mount robust pro-inflammatory respiratory burst antimicrobial responses when a phagocyte encountered both pro-inflammatory and homeostatic particles, as would commonly occur within an infection site. Importantly, priming goldfish and lamprey leukocytes in an anti-inflammatory environment (pre-incubation with apoptotic cells) resulted in similar responses in both animal groups. This was particularly relevant in the Zym+AC group, where the suppression of respiratory burst responses was now observed in both goldfish and lamprey, in contrast to experiments where both particles were added at the same time. As such, our results suggest that goldfish phagocytes remain as central contributors to the resolution phase of inflammation, even though they showcased an improved ability to induce strong antimicrobial inflammatory responses.

Following an infectious challenge, pathogen load and the number of apoptotic cells vary at each point along the inflammatory process. By altering the density of zymosan to apoptotic cells we were able to mimic this natural progression. Analysis of ROS production among mixed cell populations allowed us to assess the broader impact of phagocyte responses on the total leukocyte pool. Goldfish leukocytes required three times the number of apoptotic cells to zymosan to return to basal levels of ROS, whereas phagocytes from the sea lamprey reached basal levels when zymosan outnumbered apoptotic cells three to one. Consistent with an increased capacity for antimicrobial pro-inflammatory responses in goldfish, our results suggested a reduced sensitivity of their phagocytes to apoptotic cell homeostatic signals, and a greater potency for ROS production compared to lamprey phagocytes following zymosan stimulation. It remains to be determined if these features are shared across the range of potential pathogenic challenges (bacterial, fungal, viral, parasitic) and whether they are consistent across all phagocyte subsets. It is possible that each phagocyte population (e.g. macrophage, monocyte, neutrophil) may display distinct abilities to take up zymosan or apoptotic cells, or that the capacity of apoptotic cells to inhibit the ROS potential for each phagocyte population is different. As such, the overall capacity to elicit or inhibit ROS responses may depend on the type of phagocyte that is primarily present at that site of infection or injury, and their relative contributions to this control. Similarly, it remains to be determined if these features are part of a broadly used strategy for the regulation of phagocyte-driven inflammatory processes beyond ROS antimicrobial responses.

A reduced sensitivity of goldfish phagocytes to apoptotic cells coupled to greater potency for ROS production would presumably translate into increased efficacy for killing of invading pathogens by respiratory burst responses. However, a decreased efficacy in apoptotic cell-driven phagocyte mechanisms that attenuate this pro-inflammatory process could come at a cost unless complementary regulatory strategies are developed to ensure continued maintenance of host integrity. Collectively, our results suggest an evolving contribution of intrinsic phagocyte mechanisms to control of inflammation, and illustrate one effective strategy that allows for increased responsiveness against invading pathogens while ensuring continued participation in its resolution phase. Importantly, future studies should expand on the contributions of phylogeny and ontogeny to the relative sensitivities of phagocytes to pro-inflammatory and homeostatic signals. Among others, differing life cycles pose unique physiological challenges that drive the development of novel strategies for inflammation control. For example, during the first 4–6 years of their life, larval sea lampreys burrow in tributary sediment where they interact with their environment as blind filter feeders [40]. Subsequently, these lampreys undergo an 8-month metamorphosis period, where tissue loss and reorganization is accompanied by extensive cell death [41]. Finally, in their 12-month adult stage, the parasitic nature of adult lampreys is likely to set unique requirements for induction and control of inflammatory reactions, given that a compromise must be struck to allow for continued surveillance of natural infections while ensuring that sustained immune competence does not impinge on the fragile relationship between the parasite and its host.

## Supporting Information

Figure S1 **Comparative analysis of goldfish and lamprey primary leukocyte phagocytosis.** (A) Goldfish primary kidney leukocytes (PKLs) or lamprey primary typhlosole leukocytes (PTLs) were plated in a 6-well plate and incubated with 3 µm latex beads at the indicated concentrations for the specified times. Phagocytosis was quantified by light microscopy at 100x magnification. Phagocytic index represents the average number of beads internalized per phagocytic cell in the sample. (B) Representative images are of positive phagocytosis (beads marked with x) and surface bound beads at 100x magnification. For all n = 4 animals examined over a minimum of two independent experiments, * p<0.05 and ** p<0.01.
(TIF)

Figure S2 **Kinetics of goldfish PKL and Lamprey PTL activation as measured by ROS production.** Goldfish PKL and lamprey PTL were incubated with zymosan (5∶1 ratio) for the indicated times. Respiratory burst was measured in the total PKL and PTL population. For all n = 4, examined over a minimum of two independent experiments. * p<0.05 and ** p<0.01 compared to 0 h.
(TIF)

Figure S3 **Gating strategy for cell subpopulations isolated from hematopoietic tissues.** (A) Primary hematopoietic leukocytes isolated from goldfish kidney and representative images of cells from within each gate. (B) Primary hematopoietic leukocytes isolated from lamprey typhlosole and representative images of cells from within each gate. Cell populations were determined based on internal complexity (dark field) and area. L- lymphocytes; M/G- monocytes and granulocytes; Mac- macrophage.
(TIF)
