# Supplementary figures and images for: Evolutionary Conservation of Divergent Pro-Inflammatory and Homeostatic Responses in Lamprey Phagocytes
Source: PLoS One. 2014 Jan 20;9(1):e86255. doi: 10.1371/journal.pone.0086255 (PMC3896464; doi:10.1371/journal.pone.0086255)

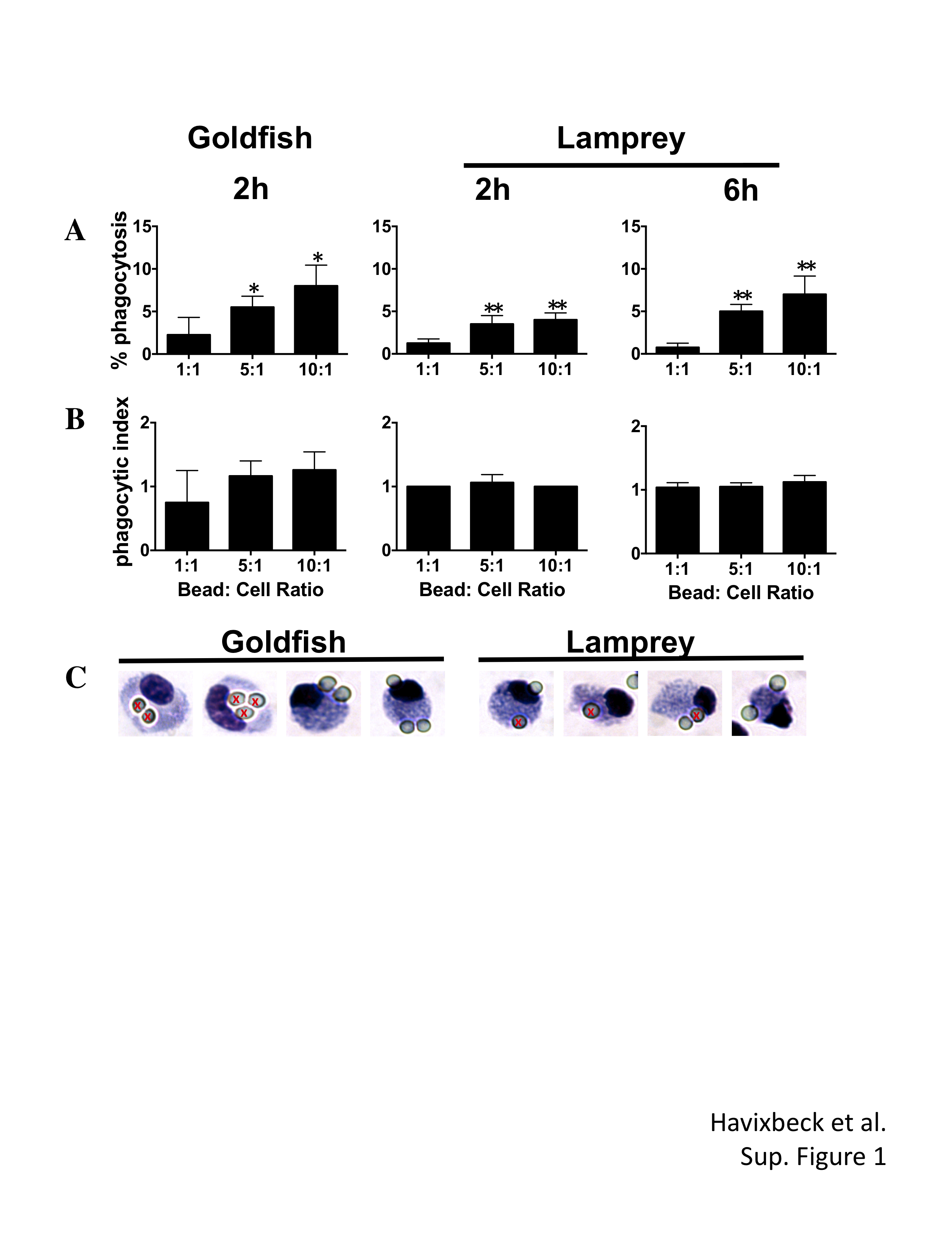

Supplement: Figure S1 — Comparative analysis of goldfish and lamprey primary leukocyte phagocytosis. (A) Goldfish primary kidney leukocytes (PKLs) or lamprey primary typhlosole leukocytes (PTLs) were plated in a 6-well plate and incubated with 3 µm latex beads at the indicated concentrations for the specified times. Phagocytosis was quantified by light microscopy at 100x magnification. Phagocytic index represents the average number of beads internalized per phagocytic cell in the sample. (B) Representative images are of positive phagocytosis (beads marked with x) and surface bound beads at 100x magnification. For all n = 4 animals examined over a minimum of two independent experiments, * p<0.05 and ** p<0.01. (TIF) [file pone.0086255.s001.tif]

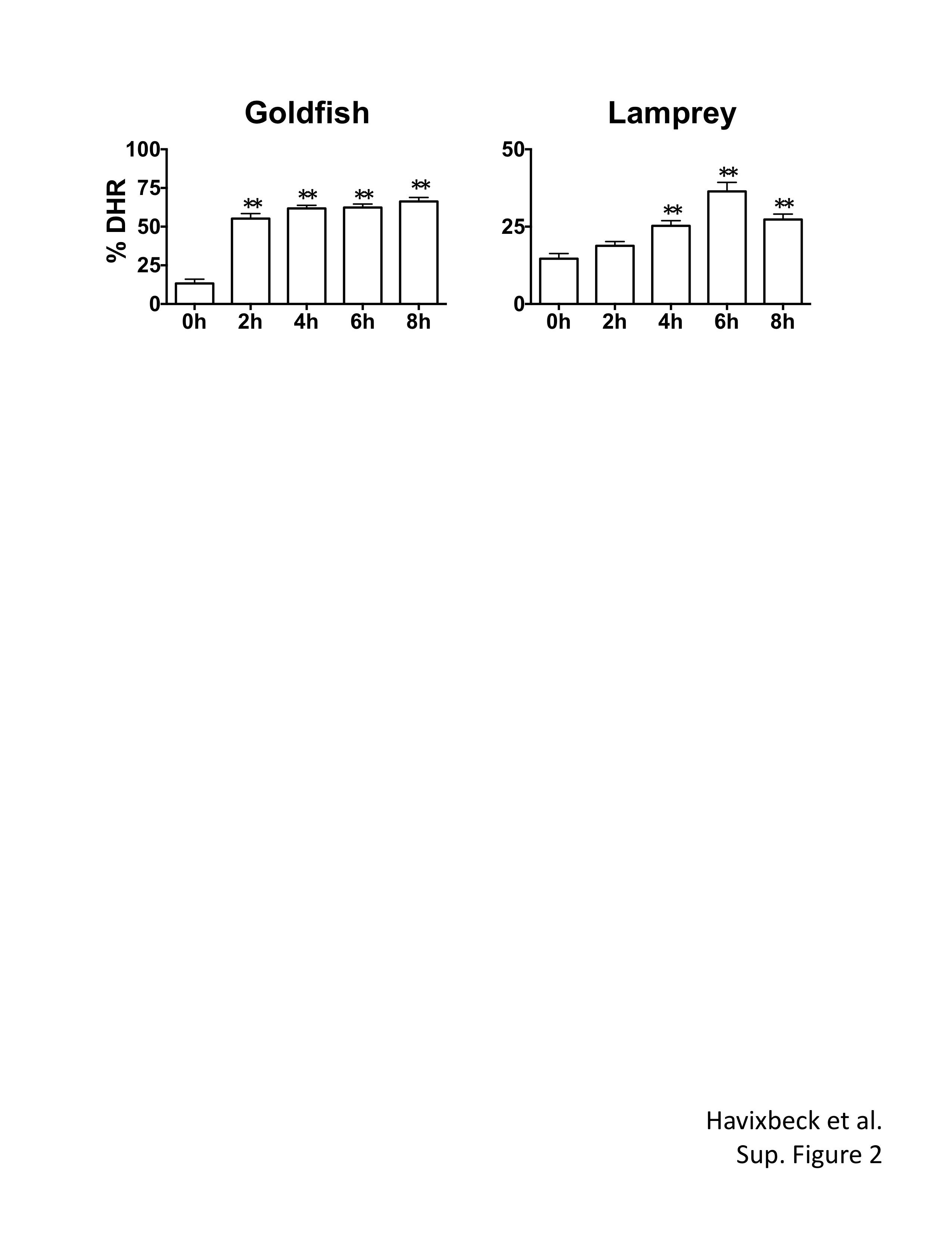

Supplement: Figure S2 — Kinetics of goldfish PKL and Lamprey PTL activation as measured by ROS production. Goldfish PKL and lamprey PTL were incubated with zymosan (5∶1 ratio) for the indicated times. Respiratory burst was measured in the total PKL and PTL population. For all n = 4, examined over a minimum of two independent experiments. * p<0.05 and ** p<0.01 compared to 0 h. (TIF) [file pone.0086255.s002.tif]

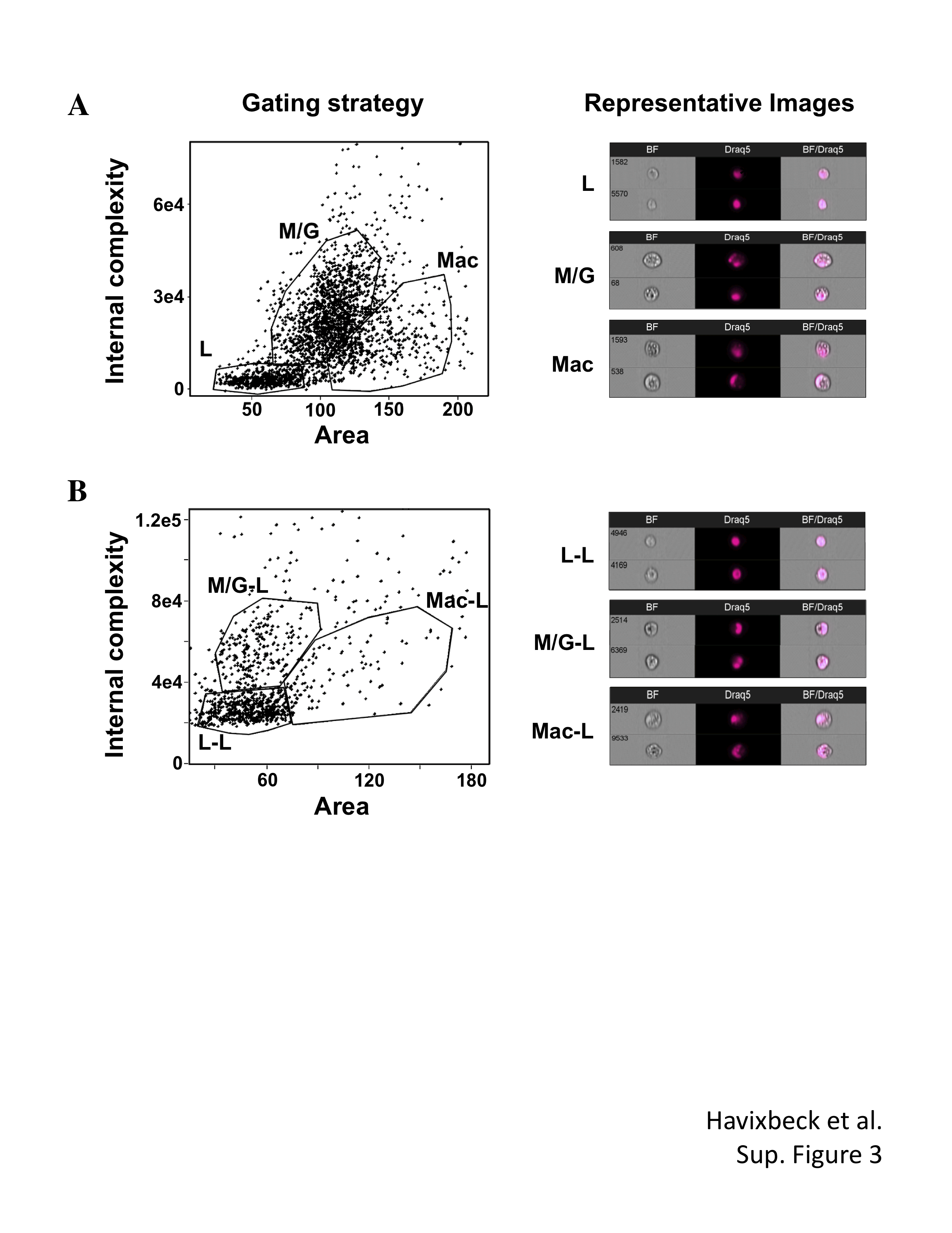

Supplement: Figure S3 — Gating strategy for cell subpopulations isolated from hematopoietic tissues. (A) Primary hematopoietic leukocytes isolated from goldfish kidney and representative images of cells from within each gate. (B) Primary hematopoietic leukocytes isolated from lamprey typhlosole and representative images of cells from within each gate. Cell populations were determined based on internal complexity (dark field) and area. L- lymphocytes; M/G- monocytes and granulocytes; Mac- macrophage. (TIF) [file pone.0086255.s003.tif]
